# A New Era in Diagnosis: From Biomarkers to Artificial Intelligence

**DOI:** 10.3390/diagnostics16101526

**Published:** 2026-05-18

**Authors:** Tudor Drugan, Daniel Leucuța

**Affiliations:** Department of Medical Informatics and Biostatistics, Faculty of Medicine, Iuliu Hațieganu University of Medicine and Pharmacy Cluj-Napoca, 6 Pasteur, 400349 Cluj-Napoca, Romania; dleucuta@umfcluj.ro

## 1. Introduction

The purpose of this Special Issue, A New Era in Diagnosis: From Biomarkers to Artificial Intelligence, was to report advancements and offer opportunities for contributions at the intersection of biomarker discovery, artificial intelligence (AI), and clinical utility.

The convergence of medical research and artificial intelligence (AI) has marked a historic inflection point, paving the way for what may be termed “high-performance medicine” [1]. Among the most transformative innovations in this domain are Neural Networks (NNs), which excel at identifying complex patterns in large-scale visual datasets, and Large Language Models (LLMs), Transformer-based architectures that are redefining natural language processing [2,3]. These developments underpin the rationale for the present special issue.

The ability of deep learning architectures to extract hierarchical features from raw, non-linear data has surpassed the limitations of classical medical analysis, enabling high-impact applications in imaging and oncology [2].

Dermatology and Radiology: Convolutional neural networks (CNNs) have achieved a level of diagnostic sophistication that is equal to or beyond that of human professionals. A landmark work by Esteva et al. showed that a CNN model trained retrospectively on a large dataset of 129,450 clinical photos achieved skin cancer classification performance comparable to that of 21 board-certified dermatologists [4]. Additionally, in thoracic radiology, the application of algorithms such as CheXNet has reached and exceeded the accuracy of radiologists in identifying pneumonia from huge collections of chest X-rays, enhancing the triage of severe patients [5].

Digital Pathology and Genetic Markers The use of deep learning approaches for whole-slide histopathology image processing has significantly improved the diagnostic pipelines for cancers such as prostate and breast malignancies. Specifically, these algorithms can robustly estimate the tumour differentiation grades (e.g., Gleason score) and so can remove the inter-observer variability which is often seen among pathologists [6]. AI models can also recognise morphological features indicative of certain genetic mutations. A recent example is the prediction of mechanisms of treatment resistance mediated by KRAS mutations by integrating computational chemistry and machine learning methodologies [7].

Workflow Optimization: Healthcare systems have increasingly adopted AI algorithms not only for diagnostic purposes but also for operational optimization. Neural networks can assess noise levels in medical images, reconstruct three-dimensional anatomical structures, and automatically flag high-risk regions for clinician review, thereby supporting stable hospital management despite chronic shortages of qualified personnel [8].

Traditionally, the processing of longitudinal clinical archives represented by Electronic Health Records (EHRs) has been severely hindered by the lack of structural uniformity, as a substantial proportion of medical information remains embedded in unstructured clinical narratives [9,10]. LLMs address this limitation through their generative capabilities, enabling contextual understanding and synthesis of specialized biomedical language [11].

Contemporary LLMs have proven to be powerful tools for analyzing patient trajectories. A recent controlled study evaluated LLM-supported Clinical Decision Support Systems (CDSS) designed to assist multidisciplinary tumor boards. The findings revealed a significant reduction in the time required to assemble and synthesize clinical reports (from an average of 8 min and 47 s to 6 min and 55 s; *p* < 0.001), while simultaneously improving the completeness and accuracy of critical patient data extracted from medical records [12].

Pharmacotherapy Assistance and Malpractice Prevention:

In a rigorous analysis of prescribing errors across 16 distinct clinical specialties, the use of LLMs as “co-pilots” integrated into clinical pharmacy workflows substantially improved patient safety. AI-assisted teams detected 1.5 times more dangerous drug interactions and prescribing errors compared to standard, non-assisted processes [13].

While they can converse and are therefore suited for medical education and virtual case-based learning, AI systems have important limits that should not be ignored [14]:

1. Algorithmic Hallucinations: AI models (especially generative) have an intrinsic risk of producing erroneous medical statements that are syntactically reasonable nonetheless [11,14]. This requires a tight anchoring of LLMs in scientifically verified datasets before being deployed in patient-facing applications.
2. Network Opacity (“Black Box” Problem): The logical traceability of a proposed diagnosis is very difficult in typical deep learning systems, which is a large obstacle to clinician trust and ethical accountability [9,10].
3. Systemic Bias and Patient Rights: Algorithms are inevitably prone to the demographic biases found in training data. Additionally, there are substantial legal and privacy concerns with using real patient clinical records for LLM pretraining.

## 2. Brief Overview of This Special Number

A number of submitted papers approach improving classification and prediction of diseases by combining diverse sources of biological data, such as genomic, transcriptomic, metabolomic, and microbiomic using multimodal and multi-omics methods. This approach helps getting a closer representation of the complexity of diseases.

Other articles in this issue report the use of machine learning to imaging, electrophysiology, speech, sleep, and other types of medical data. The included articles show encouraging results in a variety of clinical settings, such as neurology, psychiatry, infectious illness, cancer, and cognitive evaluation. The models have become more capable of extracting medically relevant information from data types that are difficult to analyze using traditional analytical techniques.

Diagnostic accuracy is very important, but it must be also accompanied by methodological rigor, transparent validation, and explainability. This issue contains several studies that explicitly address these concerns using privacy-preserving methods, external validation techniques, and explainable AI methodologies.

In addition, the issue includes studies examining the role of large language models in diagnostic reasoning, methodological assessment and guideline concordance. These papers illustrate the potential but also present the limits of new generative AI medical technologies that can help the field of diagnostic.

Overall, the papers published in this Special Issue show that diagnostics is in transformation, which includes a combination of biomarkers, multimodal data integration, and advanced computational methods. This evolution can help improving the accuracy of diagnoses, speed up the search for new biomarkers, and offer ways for more individualized treatment plans.

We hope that this Special Issue will encourage more research in this quickly developing domain and help create more precise, easily understood, and therapeutically useful diagnostic methods.

## 3. Conclusions

The academic response to the fragmentation of technology today is increasingly mirrored in the vision of a new generation of systems: Generalist Medical AI (GMAI) [15]. GMAI foundation models will be multimodal, capable of integrating and analysing radiographic images, serological data, tumour genetic sequences, and clinical discourse simultaneously, a departure from present models trained on specific domains (e.g., retinal imaging or text interpretation). Such a generalist approach would significantly minimise the need for massive manually labelled datasets and enable more fine-grained medical reasoning [15].

Deep neural networks and massive language models are altering the decision-making fabric of healthcare systems with their capabilities. Deep learning algorithms, on one hand, are pushing the limits of imaging-based diagnosis beyond human sensory perception and, on the other hand, are becoming a core tool for oncological screening. Conversely, LLMs offer structure.
